# Pathological Roles of Neutrophil-Mediated Inflammation in Asthma and Its Potential for Therapy as a Target

**DOI:** 10.1155/2017/3743048

**Published:** 2017-11-22

**Authors:** Han Gao, Songmin Ying, Yuanrong Dai

**Affiliations:** Department of Respiratory Medicine, The Second Affiliated Hospital and Yuying Children's Hospital of Wenzhou Medical University, Wenzhou, Zhejiang, China

## Abstract

Asthma is a chronic inflammatory disease that undermines the airways. It is caused by dysfunction of various types of cells, as well as cellular components, and is characterized by recruitment of inflammatory cells, bronchial hyperreactivity, mucus production, and airway remodelling and narrowing. It has commonly been considered that airway inflammation is caused by the Th2 immune response, or eosinophilia, which is a hallmark of bronchial asthma pathogenesis. Some patients display a neutrophil-dominant presentation and are characterized with low (or even absent) Th2 cytokines. In recent years, increasing evidence has also suggested that neutrophils play a key role in the development of certain subtypes of asthma. This review discusses neutrophils in asthma and potentially related targeted therapies.

## 1. Introduction

Asthma is a heterogeneous disease involving a range of cell types, including eosinophils, mast cells, T lymphocytes, neutrophils, smooth muscle cells, and airway epithelial cells, as well as various cellular components. Based on the type of inflammatory cells in sputum, asthma can be divided into four phenotypes: these are eosinophilic asthma, neutrophilic asthma, mixed granulocytic asthma, and paucigranulocytic asthma. Research has shown that neutrophilic airway inflammation occurs most frequently in steroid-insensitive asthma [1], severe asthma [2], acute exacerbation of asthma [3], and occupational asthma [4]. It was also found that persistent neutrophil inflammation occurs in some mild-to-moderate types of asthma [5]. Here, we continue to discuss the pathological effects of neutrophilic inflammation in asthma, focusing on cytokine/chemokine release and the pathogenic results of airway obstruction, hyperresponsiveness (AHR), and smooth muscle cell remodelling. We also assess the development of targeted therapies for asthma.

## 2. Regulation of Neutrophil Migration and Infiltration in Asthma

**Figure 1:**
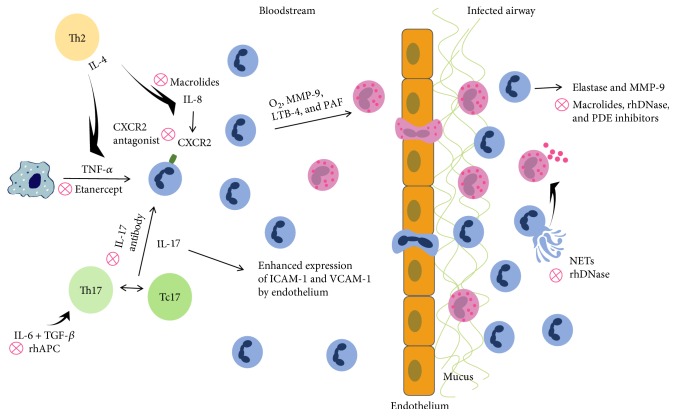
Schematic demonstration of neutrophil migration and infiltration in allergic inflammatory response.

Interleukin- (IL-) 8 was found to be elevated in asthmatic patients. As one of the chemokines for neutrophils, IL-8 may promote the migration of neutrophils to the site of inflammation [6]. Previous studies have shown that the neutrophil recruitment induced by IL-8 increases the release of O_2_, matrix metalloproteinase-9 (MMP-9), leukotrienes-4 (LTB-4), and platelet-activating factor (PAF) (Figure 1). This results in transmural movement and accumulation of eosinophils in the airways [7]. Hosoki et al. examined 48 kinds of cytokines and chemokines obtained by bronchoalveolar lavage of controlled and uncontrolled asthma patients. They found elevated levels of IL-8 and neutrophils in uncontrolled asthma patients compared to controlled asthma patients, suggesting a potential diagnostic marker for an uncontrolled state of this disease [8].

Interleukin- (IL-) 4 expression is significantly increased in asthmatic patients [9]. Lavoie-Lamoureux et al. concluded that IL-4, a cytokine predominantly secreted by Th2 cells, may have a role in activating neutrophils during allergic inflammation by regulating the release of neutrophil chemokines and cytokines. For example, IL-4 may act to increase the secretion of IL-8 and tumor necrosis factor- (TNF-) *α* (Figure 1) and thus inhibit the secretion of IL-1*β* [10]. This study also showed that IL-4 regulates the mRNA expression levels of IL-8, TNF-*α*, and IL-1*β* in neutrophils. This correlates with the severity of the disease, antigen exposure, and intracellular pathways involved in the pathophysiological processes. Related studies have also reached similar conclusions [11].

Eosinophilic asthma correlates with Th2-associated inflammation and the maintenance of an allergic reaction. Meanwhile, neutrophilic asthma is more strongly associated with the presence of Th17 cells [12]. The Th17 subpopulation is one of the subtypes of CD4+ T lymphocytes and mainly secretes IL-17A, IL-17F, and IL-22. IL-17 levels have been found to be elevated in the bronchial tissues [13], sputum [14], serum [15], and bronchoalveolar lavage fluids [16] in patients with asthma. IL-17 not only induces the secretion of granulocyte colony-stimulating factor (G-CSF) from macrophages and fibroblast cells to promote the differentiation of CD34 progenitor cells into neutrophils but also increases mucus secretion as a result of airway mucus metaplasia and promotes the activation of macrophages and fibroblasts, resulting in airway remodelling [12]. The studies [17] conducted by Roussel and colleagues found that IL-17 activates the production of chemokines, such as CXCL8, in the pulmonary vascular endothelial cells. This attracts a large number of neutrophils to inflammatory locations. Furthermore, IL-17 induces endothelial cells to secrete adhesion molecules, such as vascular cell adhesion molecule- (VCAM-) 1 and intercellular cell adhesion molecule- (ICAM-) 1, through a p38/MAPK-dependent pathway. This results in the infiltration and adhesion of neutrophils at sites of inflammation in asthma patients. Furthermore, Huber et al. showed that T cytotoxic (Tc) 17 cells promote the differentiation of Th17 cells via direct cell contact in an animal model of autoimmune meningitis. Tc17 cells also influence inflammation in its initial stages by enhancing the pathogenicity of Th17 cells [18]. Similar conclusions by Li et al. indicate that Tc17 and Th17 cells are enriched in the peripheral blood of asthmatics (and the lungs and spleens of mouse models of asthma), showing that Tc17 and Th17 cells might contribute directly to asthma pathogenesis [19] (Figure 1).

Other studies have found that IL-6 facilitates bone marrow to release polymorphonuclear leukocyte (PMN) into peripheral blood [20], which increases the binding of PMN to vascular endothelial cells [21], inhibiting spontaneous apoptosis in neutrophils [22]. In contrast, neutrophils may produce IL-6 via the ERKl/2 and p38/MAPK signaling pathways, which are activated by C5a [23]. The elevated IL-6 levels in asthmatic patients [24] may result in an inflammatory response, indicated by the decreased growth of fibroblasts and epithelial cells, in addition to impaired regeneration of tissues damaged by the allergic airway inflammation [25]. IL-6 in combination with transforming growth factor (TGF) might induce the production of Th17 cells (Figure 1) and inhibit the differentiation of Treg cells from naïve T cells [26]. IL-6 levels are negatively correlated with forced expiratory volume in one second [24], which is a key indicator of lung function during the diagnosis and treatment of asthma.

Another study found that bronchial epithelial cells cultured in vitro produce a variety of neutrophil-related chemokines, including IL-8, and a granulocyte-macrophage colony-stimulating factor (GM-CSF), TNF-*α*, and LTB-4. They also produce epidermal growth factor (EGF), which is a regulator of airway mucosal pathology in bronchial asthma [27]. As stated by Uddin et al., EGF secreted from airway epithelial cells in asthmatics may contribute to neutrophil recruitment. This likely involves class IB PI(3)Kc isoform signaling and inflammatory responses [28]. Besides functioning as a proinflammatory factor, EGF contributes to airway wall remodelling [29].

Recently, the roles of neutrophil autophagy and extracellular DNA traps (NETs) in asthma have been explored. High levels of autophagy and NETs were reported in sputum granulocytes, peripheral blood cells, and bronchoalveolar fluids in atopic asthmatics [30, 31]. Autophagy impairs the function of cellular organelles and proteins, delivering them to lysosomes for degradation in order to maintain homeostasis [32]. Reactive oxygen species released from neutrophils and microbial infections can exacerbate asthma and may modulate autophagy in polynuclear neutrophils and other cells [33, 34]. Numerous cytokines and microproteins involved in the production of NETs have been identified, and this process can be triggered by the extracellular release of decondensed chromatin [35]. Beyond that, autophagy and superoxide production can affect the formation of NETs in allergic asthma airways [36]. In recent studies, researchers isolated autophagy-related gene polymorphisms, associated with neutrophilic inflammatory and serum IL-8 levels [37]. Autophagy and NETs were found to damage the airway epithelium and irritate eosinophil degranulation (Figure 1) in airway epithelial cells and peripheral blood, which led to uncontrolled asthma [38]. At present, it is unknown how the remnants of neutrophils and NETs are cleared from airway tissues.

Baines et al. found that the expressions of *α*-defensins, neutrophil cathepsin G, and neutrophil elastase genes are significantly increased in neutrophilic asthma, and these indicators correlate with the activation and aggregation of neutrophils in patients with this type of asthma. This evokes a new strategy for identifying neutrophilic asthma on the basis of gene expression [39]. Another study [40] found that *α*-defensins may harm lung epithelial cells by adhering to the membrane surface via binding to serine protease inhibitor family members, such as *α*-protease inhibitor. Moreover, because of changes in the biological characteristics of defensins, reduced antibacterial activity, and the maintenance of proinflammatory properties resulting from ADP glycosylation of *α*-defensins, asthma patients are more vulnerable to infections.

S100A9 belongs to a large Ca^2+^-binding protein family, which has a wide range of functions. S100 is related to S100A9 and neutrophil inflammation and has proinflammatory effects that activate neutrophil chemokines, induce acute neutrophil responses [41], and increase IL-8 production [42]. S100A9 levels are significantly higher in uncontrolled neutrophilic asthma compared to uncontrolled eosinophilic asthma, chronic obstructive pulmonary disease, and controlled asthma. Sputum S100A9 levels may represent a biomarker for severe neutrophilic uncontrolled asthma [43]. Further research is required to understand the function of S100A9 in severe asthma.

Descriptions of neutrophil migration characteristics using microfluidic chips have been used for diagnostics [44]. An in vivo-like neutrophil transendothelial migration model [45] was fabricated as a microfluidic platform, as it was found that the concentration of neutrophils with various amounts of chemokines has synergistic effects. After increasing chemokine concentrations, more neutrophils moved to the chemoattractant sources. We found that endothelial cells play a crucial role in promoting morphological changes in neutrophils and upregulating the expression of related adhesion factors. The successful evaluation of neutrophil chemotaxis in patients with chronic obstructive pulmonary disease (COPD) utilizing the microfluidic system is an exciting advance [46]. We look forward to future studies using these microfluidic systems, as they might be effectively adapted for neutrophilic asthma diagnostics and therapeutic efficacy assessments.

## 3. Imbalances in Apoptosis and Survival in Asthma

The initial response to asthma occurs when IgE is combined with Fc*ε*R I on the cell surface, leading mast cells to release arachidonic acid metabolites, cytokines, and chemokines by degranulation, resulting in a series of inflammatory responses to asthma [47, 48]. A large number of polymorphonuclear leukocytes were detected in the sputum of asthmatic patients. This may be associated with the prolonged survival of leukocytes mediated by IgE expression, which can inhibit apoptosis by increasing the expression of myeloid cell leukemia- (MCL-) 1 protein and maintaining the integrity of the cell membrane. The prosurvival activity observed in neutrophils may correlate with the severity of asthma. This is evidenced by the observation that MCL-1 protein decreases the transformation activity of mitochondria and the function of cytochrome C by binding Bcl-2-associated X protein (BAX) [49]. However, Mora et al. suggested that serum IgE levels had no effect on the expression of Fc*ε*R I on neutrophils [50]. As previous studies have shown, GM-CSF inhibits neutrophil apoptosis via multiple class I PI(3)Ks [28]. Unsurprisingly, neutrophil apoptosis is a highly regulated process. Recognized “survival factors” include lipopolysaccharide, granulocyte-macrophage colony-stimulating factor, type I and II interferons, survivin, proteolytic enzymes, hypoxia, and glucocorticosteroids. With regard to signaling pathways in neutrophil survival, the PI3-kinase/AKT, NF-*κ*B, HIF-1a, and MCL-1 pathways all seem to play substantial and context-specific roles [51–56]. Any or all of these mediators may contribute to an apoptosis-resistant granulocyte phenotype, which, if operational in the airway walls of patients with asthma, might severely impede neutrophil clearance.

## 4. Relationship between Neutrophilic Inflammation and Airway Remodelling

Reversible airway obstruction (from natural remission or treatment) is universally acknowledged to be one of the hallmarks of asthma pathology. Nevertheless, there is increasing evidence that asthma can cause irreversible airway structural changes. These pathological features are collectively referred to as airway remodelling.

Oncostatin M (OSM), which is locally produced in neutrophils and macrophages, is a cytokine belonging to the IL-6 family and promotes fibrosis in many diseases. For example, OSM stimulates the growth of mouse synovial fibroblasts and human dermal fibroblasts [57, 58]. In asthmatic patients with irreversible airflow obstruction, Simpson et al. found that the secretion of OSM from neutrophils increases markedly after exposure to a high level of lipopolysaccharides [59]. Fibroblasts are the main source of collagen. OSM binds the promoter of collagen *α*2, resulting in the proliferation of human lung fibroblasts, inhibition of neutrophil clearance, and induction of collagen production [60]. OSM upregulated the expression of hepatocyte growth factor (HGF) in human lung fibroblasts via the mitogen-activated protein kinase (MAPK) pathway. HGF is a key factor secreted by fibroblasts and is involved in airway epithelial remodelling [57]. In animal models, OSM stimulates the secretion of vascular endothelial growth factors, which induce vascular reconstruction [61] and provide a material basis for the reconstruction of the tracheal wall. These studies support the hypothesis that OSM promotes continual repair of the airway epithelium, which leads to airway structural changes such as subepithelial basement thickening.

MMP-9, an enzyme secreted from neutrophils in a time- and dose-dependent manner, promotes the production of Ags and anti-IgE antibodies. Ventura and his partners believe that neutrophils not only cause allergic inflammation but also are involved in airway remodelling in asthmatic patients through the release of MMP-9 [62] (Figure 1).

It is worth mentioning that exosomes, which are released from a variety of cells in the form of vesicles, are involved in the regulation of immune reactions and various stimulus responses. In patients with asthma and in healthy individuals, exosomes in bronchoalveolar lavage fluid have multiple functions, such as stimulating airway epithelial cells to produce LTC4 and increasing the expression of cytokines involved in inflammatory responses [63]. Vargas et al. found that exosomes secreted from neutrophils regulate apoptosis and proliferation in the airway smooth muscle cells. This helps mediate airway remodelling in asthma patients [64].

YKL-40 is secreted by neutrophils as an inflammatory factor involved in asthma's inflammatory response. Tang et al. determined the level of YKL-40 in asthmatic patients is elevated, especially during the acute exacerbation of asthma [65]. Lee et al. pointed out that YKL-40 is closely related to the type Th2 inflammatory reaction [66]. In the course of asthma progression, YKL-40 also plays an important role in airway remodelling. Chupp et al. confirmed that local airway and serum YKL-40 were positively related to subepithelial basement membranes in asthma patients through a large, multicenter clinical study [67]. Bara et al. found that YKL-40 can promote proliferation, migration, and antiapoptosis of bronchia smooth muscle cells through a variety of signaling pathways [68]. Further research discovered that YKL-40 promotes IL-8 expression via the MAPK and NF-*κ*B signal pathway, indirectly promoting the bronchial smooth muscle cell proliferation and migration [69]. This study finds that YKL-40 can estimate the development and severity of asthma. However, changes in these levels exist in other systemic diseases, and the diagnosis of asthma is lacking in specificity, requiring close integration with clinical and other ancillary tests, while excluding the effect of other diseases. It is unknown whether YKL-40 plays a role in the prognosis of asthma and becomes a new therapeutic target. More research is needed to support YKL-40 for clinical use.

Other cytokines involved in neutrophils for tissue injury and repair are neutrophil elastase, myeloperoxidase (MPO), bFGF, PDGF, VEGF, oxidation products, and serine neutral protease. These mediators are involved in epithelial damage, fibrosis, and angiogenesis [70–75].

## 5. Neutrophil and Airway Hyperresponsiveness

Neutrophil elastase, a protease released from activated neutrophils, has been thought to be involved in the etiology of asthma because it seems to induce airway mucus gland hyperplasia, mucus secretion, and airway smooth muscle cell proliferation [39]. Studies show that decreased contractility of the small airways and baseline patency are attributable to proteolysis and the analysis of neutrophil elastase in smokers, nonsmokers, and COPD patients [76]. One study found that the specific neutrophil elastase inhibitor, sivelestat, preserves the protective effects of airway inflammation by reducing AHR expression, eosinophil numbers, and Th2 cytokine levels. It also influences goblet cell metaplasia [77]. Neutrophil elastase can therefore partially induce AHR in asthmatics.

## 6. Possible Mechanisms of Chronic Airway Obstruction Mediated by Neutrophilic Inflammation

Choi et al. compared the clinical features and physiological parameters of 77 refractory asthma patients with or without persistent airway obstruction and evaluated the cellular composition of their sputum [78]. These data were compared to those from patients undergoing hormone therapy, who did not have persistent airway obstruction. They had to use a higher dose, with persistent airway obstruction in asthma patients involving a longer duration and more serious symptoms. Neutrophilic inflammation occurs mainly with persistent airway obstruction, while eosinophilic inflammation occurs without any persistent airway obstruction. There is a strong link between asthmatic neutrophilic inflammation and progression of persistent airflow limitation.

IL-8 is a potential stimulator of NET in asthma airways [79]. NET production induced by IL-8 is consistent with a decreased FEV1/FVC ratio, while the FEV1% or FVC% of predicted values displays no significant changes [38]. These findings indicate that NET may be associated with AHR in asthma.

To summarize, the abovementioned mechanisms suggest that neutrophils are involved in the migration and infiltration through a range of inflammatory factors with asthma. Neutrophil apoptosis defects cause an excessive inflammatory response. Activated neutrophils can result in AHR, bronchospasm, stenosis, lung tissue damage, gland hypersecretion, and airway remodelling, leading to irreversible alterations in airway structure. The majority of patients with bronchial asthma had poor control and eventually developed severe asthma. It has been mentioned that severe asthma is largely caused by neutrophil inflammation and is speculated that neutrophil inflammatory reaction may play an important role in the pathogenesis of severe asthma. The current study of neutrophils in asthmatics is predominantly from induced sputum or bronchoalveolar lavage fluid, but few studies have been done on the neutrophil count in the airway wall. It is unclear whether neutrophils play a crucial role or have only bystander effects in the pathogenesis of severe asthma. In addition, neutrophil asthma is the result of hormone therapy, as glucocorticoid treatment can easily suppress eosinophilic inflammation rather than neutrophil inflammation. With inhaled corticosteroid treatment, the proportion of eosinophils decreased significantly, and the proportion of neutrophils increased [80]. Nguyen et al. reported that in mildly stable patients, eosinophils were significantly decreased 1 month after inhaled corticosteroid treatment, but the proportion of neutrophils did not change [81]. This may contribute to errors in determining the inflammatory phenotype of asthma. At present, inhaled corticosteroid is one of the basic drugs for treatment of asthma. If this standpoint is confirmed, this would deserve further investigation as to whether glucocorticoids play a controlling role or act as a progressive irritant in the treatment of some asthmatics.

## 7. Microbial Infection and Neutrophil Inflammation

It has been shown that the lower respiratory tract is not a completely sterile environment, especially with such a large number of microbial flora. When chronic airway inflammatory disease occurs, including asthma, the type and amount of bacterial flora in the lower respiratory tract substantially change [82]. We find that most hospitalized patients with an acute attack of bronchial asthma have respiratory infections, which aggravate the symptoms of asthma. Neutrophils, as the first line of defense against respiratory pathogens, play a critical role in the immune system.

*Human rhinovirus*, *respiratory syncytial virus*, and *influenza virus* infections are associated with increased risk of asthma. The study by Tang et al. provides evidence that neutrophils contain the immune repertoire to directly detect and respond to viruses. Viral infection can promote elastase, MMP-9, CXCL8 secreted by neutrophils in order to participate in the trajectory of asthma [83]. Green et al. analyzed the composition of airway microorganisms in patients with severe asthma at its stable stage, using induced sputum samples, confirming that *Haemophilus influenzae*, *Streptococcus pneumoniae*, and *Moraxella catarrhalis* were positively related to sputum neutrophil levels and decreased FEV1. There was no significant increase in the frequency of acute exacerbations of asthma in an estimated year [84]. Gram-positive bacterial cell wall components (such as lipoteichoic acid), negative cell membrane components (such as lipopolysaccharide), and endotoxins as pathogens associated with molecular patterns (PAMP), combined with Toll-like receptors (TLRs) on the surface of immune cells for the inflammatory cascade reaction, actually promote the production of cytokines (IL-8, IL-1, and TNF-*α*) and conversion of Th1 cells to Th17 cells. G protein coupled receptors (such as GPCR41 and GPCR43) expressed on the surface of neutrophils identify PAMP and promote neutrophil aggregation, ultimately contributing to the development of steroid resistance in neutrophilic asthma phenotypes [85–87].

We must wonder whether there are susceptible pathogens in asthmatics and whether long-term hormone therapy causes selective growth of bacterial flora. Yayan did not find a direct association between asthma and microbes after comparing the relationships among pathogens (such as bacteria, viruses, and fungi with different types of asthma) [88]. However, the authors did not assess whether early respiratory infections in children will have an impact on their future course with asthma issues. When microbes are infected, neutrophils rush to the field to fight pathogens. Glucocorticoids are involved in the mechanism of neutrophil apoptosis delay and produce persistent neutrophil inflammation. In patients with acute exacerbation of asthma, excessive neutrophil inflammation may be the cause of an uncontrollable asthma course.

## 8. Targeted Innovative Therapeutic Approaches to Neutrophilic Asthma

Despite the improved treatment strategies for asthma, there are still many patients with no significant improvement, especially those who suffer from neutrophilic asthma. These patients do not respond to corticosteroid administration. In order to alleviate the symptoms of these patients, reduce the frequency of acute exacerbations, and improve their quality of life, a targeted therapy for neutrophil-dominated inflammation is needed.

The efficacy of IL-17 antibodies has been demonstrated in other diseases related to Th17 cells. These include psoriatic arthritis [89], rheumatoid arthritis [90], and ankylosing spondylitis [91]. However, whether the IL-17 antibody is effective for asthma remains to be determined. Experiments in a mouse model of asthma suggest that IL-17 antibodies reduce the levels of eosinophils, lymphocytes, neutrophils, IL-4, IL-5, and IL-13 in bronchoalveolar lavage fluid [92]. A clinical trial indicated that the antibody for IL-17 receptor A, brodalumab, has no effect on asthma control scores, the numbers of symptom-free days, or FEV1 in patients with moderate-to-severe asthma [93]. Further studies are needed to definitively assess the efficacy of IL-17 antibodies in the treatment of asthma, especially inadequately uncontrolled neutrophilic asthma. Dopamine induces differentiation of Th17 cells by binding to the dopamine D1-like receptor (D1-R) on dendritic cells. D1-R antagonists inhibit Th17 cell production and may be a promising strategy for treatment of severe asthma when neutrophils are present [7]. Another IL-17 antibody or different patient populations may lead to different results.

By evaluating the expression of biomarker genes in patients with asthma, Baines et al. found that CLC, CPA3, and DNASE1L3 expression increased in patients with eosinophilic asthma, while IL-1A, ALPL, and CXCR2 expression increased in patients with neutrophilic asthma [94]. CXCR2, a receptor for IL-8, is involved in neutrophil chemotaxis, protease production, airway beaker cell proliferation, lung angiogenesis, collagen deposition, and airway smooth muscle contraction [95]. Related studies have been carried out on patients with asthma who were treated with CXCR2 antagonists to inhibit neutrophil infiltration. A study was conducted to investigate the effects of the CXCR1/CXCR2 antagonist, SCH527123, on the bone marrow, peripheral blood, airway neutrophil levels, and the migration of bone marrow- and peripheral blood-derived neutrophils in patients with mild allergic asthma. Compared to the patients in the placebo group, the number of neutrophils in the peripheral blood and sputum is lower in patients treated with SCH527123, but not in the bone marrow [96]. Likewise, a randomized, placebo-controlled clinical trial came to a similar conclusion [97]. After administering SCH527123 to severely affected asthma patients with neutrophils > 40%, the number of neutrophils in the sputum was reduced to 36.3%. This is accompanied by a decrease in the level of acute exacerbation and improvement of clinical symptoms, suggesting that antagonists are effective in severe asthma patients. Other CXCR2 antagonists, such as AZD8309 and SB656933, have also been developed to study their therapeutic effects on neutrophilic inflammation [98, 99].

O'Byrne et al. studied another CXCR2 inhibitor, AZD5069. Unfortunately, there was no significant difference in the effect of AZD5069 on the number of episodes of asthma exacerbations compared to placebo. The authors excluded patients with increased peripheral blood eosinophils or IgE, whose acute exacerbations were more likely to be dominated by eosinophils versus neutrophils. Patients with mild-to-moderate asthma treated with different doses of AZD5069 showed no significant improvement in lung function or asthma control questionnaire scores by 6 months, suggesting that an inadequate dose is not the reason for poor therapy. After using the maximum dose of 45 mg, peripheral blood neutrophil counts decreased but once stopped, it increased again. Perhaps, the CXCR2 antagonist AZD5069 may act as an adjunctive therapy in concert with inhaled corticosteroids and long-acting *β*2 agonists. In conclusion, CXCR2 antagonists have not been recognized for the treatment of neutrophilic asthma, and perhaps a more illuminating result will compare different CXCR2 antagonists [100].

The TNF-*α* antagonist restores glucocorticoid sensitivity in a novel mouse model of neutrophilic airway inflammation. TNF-*α* prevents glucocorticoid receptor activity in the nonpulp chamber, resulting in impaired glucocorticoid receptor dimerization and related activities. TNF-*α* inhibitors, such as etanercept, are not effective in patients suffering from neutrophil-based asthma because they are not sensitive to glucocorticoids [101]. Neutralizing TNF-*α* improves AHR and airway obstruction in severe asthma [102]. In Th17+ and neutrophil-dominant, steroid-resistant asthma, TNF-*α* inhibitors have protective functions, significantly suppressing pulmonary inflammation, improving lung compliance and reducing neutrophil cytokine secretion and neutrophil infiltration [103, 104]. The clinical effects of TNF inhibitors are not ideal. The effectiveness and security of golimumab (a human monoclonal antibody against TNF-*α*) have been tested. After its use, patients developed a greater risk of life-threatening infections and some malignancies: breast carcinoma, metastatic melanoma, B-cell lymphoma, renal cell carcinoma, cervical carcinoma, basal cell carcinoma, and colon cancer, all of which led to early discontinuation. This result warrants further study [105].

We have previously discussed the influence of neutrophil NETs on asthma and concluded that they are associated with airway hyperreactivity in asthmatic patients. The effective targeting of NET structures was applied in the therapeutic treatment for various diseases, including psoriasis [106], systemic lupus erythematosus [107], sepsis [108], thrombotic disorders [109], and human immunodeficiency virus-1 (HIV) infection [110]. Neutrophils undergo high levels of autophagy and possess elevated amounts of neutrophil extracellular trap DNA. They activate neutrophils and eosinophils, which exacerbate severe asthma, destroy airway epithelial cells, and stimulate the inflammatory reaction in airway epithelial cells and eosinophils [38]. Recent studies have shown that the use of rhDNase can reduce airway resistance, improve lung function, inhibit formation of eosinophilic extracellular traps, and halt goblet cell hyperplasia [111]. The effect of rhDNase on the neutrophil extracellular trap is currently unknown.

Recent research has demonstrated the safety and efficacy of the long-term administration of macrolides in COPD patients [112], and the study of macrolide therapies in asthma patients has also begun. Besides their antibacterial effects, macrolides reduce levels of IL-8 and the number of neutrophils in refractory neutrophilic asthma patients, which relieves airway inflammation, inhibits neutrophil aggregation, and attenuates neutrophil elastase and MMP-9 production [113–115]. Recent studies have shown that macrolides can inhibit TNF-*α*- and IL-17-induced immune responses and restrain AHR [116]. The mechanisms underlying this process involve the inhibition of cellular signal transduction pathways (such as NF-*κ*B [117] and PI3K [118]) and the restoration of histone deacetylase-2 (HDAC2) activity [119]. Long-term use of macrolide drugs increases adverse drug reactions and promotes development of antimicrobial resistance. Taking into account the adverse effects of antibiotics, the long-term use of macrolides for asthma treatment should be approached with caution.

Imbalances in blood, for example, coagulation versus anticoagulation, may aggravate airway inflammation in asthma [120]. C protein, which is synthesized by the liver, is an anticoagulant that hydrolyzes activated coagulation factor VIII and factor V while suppressing the activation of coagulation factor X and prothrombin. It also promotes fibrinolysis. Schouten et al. discovered that activated protein C decreases within 4 h after exposure to this antigen, which was determined by assaying the bronchoalveolar lavage fluid from asthmatic patients [121]. Additionally, the percentage of APC/thrombin-expressing cells in the sputum was reduced, suggesting that lower concentrations of protein C in the blood during pathogenesis of asthma might inhibit anticoagulation and overall inflammatory response [122]. It was found that activated protein C reduces the release of IL-6 from neutrophils and inhibits the expression of neutrophil chemokines. However, this has no effect on respiratory burst activity, apoptosis, or the expression of other cytokines [123]. Recombinant human activated protein C suppresses neutrophil migration [124] and degranulation of lactoferrin, which is a component of neutrophil-specific particles involved in activating eosinophils [125]. It also alleviates pulmonary damage resulting from the presence of elastase [126]. Notably, the effect of activated protein C on inflammation was not dependent on local anticoagulation [124]. This supports the hypothesis that rhAPC involves a variety of mechanisms that affect neutrophil responses, and rhAPC treatment may alleviate inflammation in allergic asthmatics by restoring the protein C pathway. Neutrophils also play a leading role in the majority of patients with severe refractory asthma and acute exacerbations. Thus, these findings bring great hope to patients who respond poorly to conventional asthma therapies.

Phosphodiesterase (PDE) inhibitors have a wide range of effects and may be useful for treating neutrophilic asthma. Early studies have shown that PDE inhibitors inactivate neutrophil elastase and MMP-9 release and also reduce the ability of neutrophils to adhere to vascular endothelial cells [127]. In an allergen provocation test, roflumilast, an oral PDE-4 inhibitor, was shown to reduce the number of neutrophils and eosinophils in sputum while alleviating bronchospasms and AHR [128]. Singh et al. studied the PDF4 inhibitor, GSK256066, which has a good inhibitory effect on both the immediate and delayed asthmatic response. These drugs are well tolerated and have less systemic exposure [129]. In recent years, studies of another PDE4 inhibitor, CHF6001, have reached similar conclusions [130]. In addition, Franciosi et al. used an inhaled preparation of an inhibitor of PDE3/PDE4, called RPL554, to test its effects on pulmonary function in asthmatic and COPD patients. This treatment was found to improve patients' symptoms, indicating it had a bronchodilatory effect. It inhibits LPS-induced neutrophil responses in healthy subjects [131]. Cumulatively, this research demonstrates PDE inhibitors are promising drugs for treatment of neutrophilic asthma.

With respect to neutrophil apoptosis, new insights have been gleaned. In particular, the Bcl-2 family has been well studied for its role in mediating apoptosis, which can be divided into two categories: One is antiapoptosis, which involves Bcl-2, Bcl-XL, and Bcl-W. The other one is the promotion of cell death, which involves Bax, Bak, and other genes. The Bcl-2 family was found to inhibit cell death in blood lymphocytes. This family was found to have similar roles in many other cell types, including neutrophils [132, 133]. In patients with steroid-insensitive asthma, glucocorticoids inhibit neutrophil apoptosis by upregulating the expression of the antiapoptotic Bcl-2 family genes [134]. Some researchers found that the expression of Bcl-2 increases in a mouse model of neutrophilic asthma and that the inflammatory response does not readily subside. This leads to a prolonged disease state, suggesting that Bcl-2 may inhibit apoptosis in neutrophilic granulocytes. After the application of the Bcl-2 inhibitors, ABT-737 or ABT-199, which bind antiapoptotic proteins, neutrophil apoptosis increases. This subsequently alleviates the inflammatory response and AHR [135]. This indicates that Bcl-2 inhibitors may be a promising therapeutic option for corticosteroid-insensitive neutrophilic asthma.

A large number of adjunctive therapies have also been studied. Theophylline inhibits neutrophil chemotaxis [136] and reverses corticosteroid resistance [137]. Long-acting beta-agonists (LABA) mitigate the inflammatory reaction mediated by eosinophils and neutrophils by inhibiting IL-8 production [138, 139]. Leukotriene receptor antagonists inhibit superoxide generation and the production of LTB-4 [140]. Statins attenuate the secretion of chemokines, such as IL-6 [141], while enhancing corticosteroid activity [142]. Research on different treatment strategies for neutrophilic inflammation is increasing. However, it is currently unknown if multidrug combination therapies have synergistic effects on the efficacy of disease treatment.

## 9. Future Work

The role of neutrophils in asthma etiology is multidimensional, and research is limited. Further study is needed to determine whether there are other effects exerted by neutrophils in this disease's development. So far, animal models of neutrophilic asthma have not yet been established. An appropriate animal model should be established based on reasonable standard guidelines, which would provide a better foundation for research on the pathogenesis, treatment, and prevention of neutrophilic asthma. In the near future, the study of pathophysiological processes underlying asthma will lead to clinical drug development, bringing hope to patients suffering from severe asthma, glucocorticoid insensitivity, acute exacerbation asthma, and occupational asthma.
